# Machine learning insights into nurse retention through job satisfaction and financial incentives

**DOI:** 10.3389/fpsyg.2026.1796483

**Published:** 2026-05-07

**Authors:** Ayşe Atılgan Sarıdoğan, Nabi Küçükgergerli, Muzaffer Ertürk, Murat Emeç, Adem Yaman

**Affiliations:** 1 Department of Accounting and Tax Applications, Çanakkale Onsekiz Mart University, Çanakkale Vocational School of Social Sciences, Çanakkale, Türkiye; 2 Department of Business Administration, Istanbul Health and Technology University, Faculty of Economics, Administrative, and Social Sciences, Istanbul, Türkiye; 3 School of Civil Aviation, Istanbul Nişantaşı University, Istanbul, Türkiye; 4 Directorate of Internal Audit (İç Denetim Direktörlüğü), Çanakkale Onsekiz Mart University, Çanakkale, Türkiye

**Keywords:** financial incentives, healthcare management, job demands-resources model, job satisfaction, machine learning, nurse retention, nursing staffing quality, predictive modeling

## Abstract

The global nursing shortage has reached a critical inflection point, where the financial sustainability of healthcare institutions is increasingly determined by their ability to maintain a stable, high-quality workforce. This study investigates the structural determinants of nurse staffing quality—operationalized as an institutional-level proxy for retention capacity—by integrating financial incentives, workload demands, and job-satisfaction metrics into an advanced machine-learning framework. Using the comprehensive CMS Provider Information dataset (*N* = 15,640 nursing facilities), we developed and validated a predictive architecture comparing Random Forest, Support Vector Machines, and Histogram-based Gradient Boosting (HGB) models. Our analysis reveals a clear hierarchy of influence: while Financial Incentives and penalties (Total Fines, importance weight: 0.083) and Job Satisfaction Proxies (QM Rating, 0.079) serve as significant secondary drivers, the primary boundaries of staffing stability are governed by Workload and capacity constraints, specifically the Number of Residents (0.309) and Number of Certified Beds (0.287). The Gradient Boosting model emerged as the superior predictive tool (Balanced Accuracy: 0.42; Macro F1: 0.41), demonstrating that institutional scale and patient volume are the dominant predictors of staffing quality ratings. These findings suggest that financial interventions alone are insufficient; sustainable nurse retention requires a dual-strategy that aligns fiscal incentives with rigorous workload management and capacity optimization. By identifying these high-impact variables and explicitly acknowledging the limitations of proxy-based operationalization, this research provides a data-driven roadmap for policymakers and healthcare executives to mitigate turnover and enhance the financial and operational resilience of nursing care systems.

## 1 Introduction

The global healthcare landscape is facing a critical shortage of nursing professionals, making nurse retention a top priority for health policymakers and hospital administrators alike. Nurse retention is not merely a human resource metric; it is a fundamental determinant of patient safety, clinical outcomes, and the overall operational sustainability of healthcare delivery systems (Shields and Ward, 2001; de Vries et al., 2023). The decision of a nurse to remain within an organization or leave the profession entirely is a complex, multifaceted phenomenon driven by an interplay of individual, organizational, and systemic factors (Cowin, 2002; Lu et al., 2005).

A primary driver of retention identified in the literature is job satisfaction. Extensive research has shown that nurses who perceive high levels of professional autonomy, receive adequate organizational support, and experience transformational leadership are significantly more likely to stay in their roles (Chato, 2024; Penconek et al., 2021; Bautista et al., 2020). Job satisfaction acts as a psychological contract between the nurse and the institution; when this satisfaction is eroded by poor workplace engagement or a lack of career growth, turnover intention inevitably rises (Waltz et al., 2020; Cowin et al., 2008; Bae, 2024). Systematic reviews have consistently highlighted that internal work well-being and work-life balance are essential for maintaining a stable nursing workforce (Alanazi et al., 2023; Bimpong et al., 2020).

However, job satisfaction cannot be viewed in isolation from workload and staffing levels. The “workload-burnout-turnover” cycle is a well-documented challenge in nursing (Dall’Ora et al., 2020; Akinyemi et al., 2022). Excessive job demands, mandatory overtime, and high patient-to-nurse ratios lead to physical and emotional exhaustion, which directly diminishes job satisfaction and triggers the intent to leave (Chato, 2024; Hairr et al., 2014). Studies focusing on acute and intensive care units emphasize that when workload exceeds a manageable threshold, the perceived quality of care declines, further demoralizing staff and accelerating attrition (Penconek et al., 2021; Hairr et al., 2014). This relationship is particularly acute in high-stress environments or conflict zones, where the demands on nursing staff are extreme (Hynes et al., 2025; Keith et al., 2020).

Furthermore, financial incentives and compensation structures play a pivotal role in the retention equation, serving as both hygiene factors and motivators. While nursing is often driven by intrinsic motivation, the impact of pay satisfaction and financial rewards on long-term commitment cannot be understated (de Vries et al., 2023; Kenny et al., 2015; Pietersen, 2005). Competitive wages and performance-based incentives have been shown to mitigate the negative effects of high workloads, especially during crises such as the COVID-19 pandemic (Waltz et al., 2020; Hynes et al., 2025; Kolokythas et al., 2025; Beaulieu et al., 2024). In many developing and developed health systems, the gap between labor force expectations and actual compensation remains a primary cause for clinical exit (Lu et al., 2005; Cowin et al., 2008; Laboso et al., 2025; Pohan et al., 2025).

Despite the wealth of qualitative and cross-sectional studies, there is a growing need for data-driven approaches to understand these dynamics at scale. Traditional statistical methods often struggle to capture the non-linear relationships between institutional quality, financial health, and staffing stability (Akinyemi et al., 2022; Lu et al., 2012). Machine Learning (ML) offers a robust framework for analyzing large-scale administrative datasets to uncover hidden patterns in nurse retention.^1^ By utilizing institutional proxies—such as staffing ratings, deficiency scores, and ownership types—ML models can provide predictive insights that help administrators proactively address staffing shortages before they reach a critical point (Richards and Kieffer, 2022; Vrbnjak et al., 2025).

Theoretical Positioning and Contribution. This study explicitly anchors its empirical analysis within the Job Demands-Resources (JD-R) model (Demerouti et al. (2001), Bakker and Demerouti, (2007)), which posits that the balance between job demands and job resources determines employee well-being and retention. We extend this framework by: (1) operationalizing retention at the institutional level through RN Staffing Rating while transparently acknowledging its proxy limitations; (2) integrating financial constraints as a moderating resource variable; and (3) employing interpretable ML to quantify the relative weight of demand versus resource factors. This approach bridges micro-level psychological theories with macro-level administrative data, offering a novel methodological contribution to workforce analytics in healthcare.

This study aims to bridge the gap between theoretical retention frameworks and practical data science. Using a comprehensive dataset of over 15,000 healthcare providers, we investigate how workload metrics and financial/organizational proxies interact to influence RN Staffing Ratings—a key indicator of an institution’s capacity to retain qualified nursing staff. By integrating the pillars of job satisfaction, workload, and financial incentives into a predictive model validated through rigorous cross-validation and robustness checks, this research contributes to a more nuanced understanding of the systemic drivers of the nursing workforce crisis (Olaniyan et al., 2023; Rana and Shakya, 2021; Sachdeva and Jain, 2025; Kolokythas et al., 2025).

## 2 Theoretical framework: integrating JD-R, workload, and satisfaction

To strengthen the linkage between our empirical findings and established theory, we explicitly map our variables onto the Job Demands-Resources (JD-R) model (39, 40). The JD-R framework provides a parsimonious yet flexible structure for understanding how organizational factors influence employee outcomes such as burnout, satisfaction, and retention.

### 2.1 Job demands: workload as a structural constraint

Within the JD-R model, job demands are conceptualized as aspects of the job that require sustained physical or psychological effort and are consequently associated with physiological and psychological costs (40). In our institutional-level analysis, we operationalize these demands through two complementary dimensions. First, facility scale and occupancy pressure are captured by the Number of Certified Beds and the Number of Residents, which serve as proxies for structural workload intensity (Chato, 2024; Dall’Ora et al., 2020). Second, staffing intensity is measured using the Adjusted and Reported RN Hours per Resident per Day; these metrics serve as direct indicators of workload distribution, with lower values indicating a higher per-nurse workload burden (Hairr et al., 2014; Wong et al., 2024). Together, these variables provide a comprehensive institutional-level representation of the job demands that shape nurse retention capacity.

Empirical evidence consistently shows that excessive demands deplete nurses’ energy reserves, triggering the health impairment pathway of the JD-R model and increasing turnover intention (Dall’Ora et al., 2020; Quesada-Puga et al., 2024). Our analysis tests whether these demand-side variables dominate predictive models of staffing quality, as theory would suggest.

### 2.2 Job resources: financial incentives and organizational support

Job resources are conceptualized as aspects of the job that help achieve work goals, reduce job demands, or stimulate personal growth (40). In our institutional-level analysis, we operationalize these resources through two complementary dimensions. First, financial and regulatory capacity is captured through Total Fines ($) and Ownership Type, which serve as proxies for an institution’s fiscal flexibility and exposure to regulatory pressure; these factors directly influence the ability to offer competitive compensation packages and invest in workforce stability (Cowin et al., 2008; Laboso et al., 2025; Terera and Ngirande, 2014). Second, the work environment is represented through Overall Rating, QM Rating, and the presence of a Resident and Family Council, which collectively reflect organizational climate, quality-oriented culture, and participatory support structures—constructs consistently identified in the literature as critical resources for fostering job satisfaction and long-term retention (Shields and Ward, 2001; Lu et al., 2012; Niskala et al., 2020). Together, these operationalizations enable a comprehensive assessment of how institutional-level job resources may buffer workload demands and support the quality of nurse staffing within the JD-R framework.

The JD-R model posits that resources can buffer the negative impact of demands and foster a motivational pathway leading to engagement and retention (40). Our analysis examines whether resource variables moderate or independently predict staffing outcomes.

### 2.3 Conceptual integration and hypothesized pathways

Our hypothesized relationships: Workload Demands exert a direct negative pressure on Staffing Quality (retention proxy). In contrast, Financial/Organizational Resources exert a direct positive influence and may buffer the effects of demand. Job Satisfaction Proxies (quality ratings) are positioned as both an outcome of the demand-resource balance and a mediating resource for retention.

## 3 Materials and methods

### 3.1 Study design and conceptual clarification of the outcome variable

This study employs a quantitative, cross-sectional research design to analyze the institutional determinants of nurse retention and staffing quality.

Conceptual Clarification: We operationalize “retention capacity” using the RN Staffing Rating, a standardized CMS metric that reflects a facility’s reported ability to maintain adequate RN coverage. While this proxy is necessary given the absence of individual-level turnover data in administrative datasets, we explicitly acknowledge its limitations: (1) it reflects reported staffing levels rather than actual turnover rates; (2) it may be influenced by reporting practices or temporary staffing adjustments; and (3) it captures institutional capacity rather than individual nurse decisions. These limitations are discussed in Section 6 (Limitations) and temper causal interpretations of our findings.

### 3.2 Data source and sample

The primary data for this analysis are derived from the Nursing Home Quality & Staffing dataset (see Footnote 1), which provides comprehensive administrative records for 15,640 healthcare providers. The unit of analysis is the individual healthcare facility. This macro-level approach enables broader geographical and organizational comparisons often missing in localized clinical studies (Akinyemi et al., 2022; Vrbnjak et al., 2025). Facilities with missing values for the primary staffing outcome were excluded, resulting in a final analytical sample of *N* = 15,183 facilities, maintaining high statistical power for machine learning applications.

### 3.3 Variable operationalization and theoretical mapping

To align empirical analysis with the JD-R framework, variables were categorized into three functional groups (Table 1).

**Table 1:** Operational definition of variables and theoretical mapping to JD-R constructs.

Category	Dataset variable	Theoretical construct	Reference
Outcome	RN Staffing Rating	Staffing Stability / Retention Proxy	(Hairr et al., 2014; Wong et al., 2024)
Workload	Number of Residents	Job Demands / Occupancy	(Chato, 2024; Dall’Ora et al., 2020)
Workload	Adjusted RN Hours	Staffing Intensity	(Hairr et al., 2014; Quesada-Puga et al., 2024)
Environment	Overall Quality Rating	Job Satisfaction / Work Climate	(Lu et al., 2012; Ayalew et al., 2019)
Environment	Resident Council	Organizational Support	(Shields and Ward, 2001; Pahlevan Sharif et al., 2022)
Financial	Ownership Type	Institutional Resources	(Cowin et al., 2008; Terera and Ngirande, 2014)
Financial	Total Fines ($)	Financial Constraint / Quality Penalty	(Laboso et al., 2025; Purwanti et al., 2025)

### 3.4 Data preprocessing and machine learning pipeline

To ensure methodological rigor and reproducibility, the data underwent a systematic preprocessing pipeline designed to address common challenges in large-scale administrative datasets. First, missing data were handled using a stratified imputation approach: continuous variables with less than 5% missingness were imputed using median values calculated within ownership-type strata, thereby preserving the underlying distributional properties across institutional categories; categorical features were subsequently one-hot encoded to ensure compatibility with machine learning algorithms. Second, normalization procedures were tailored to variable distributions and model requirements: skewed continuous variables, such as Total Fines, were log-transformed before scaling to reduce the influence of extreme values, and all features were standardized to zero mean and unit variance for the Support Vector Machine and Histogram-based Gradient Boosting models, whereas tree-based models utilized raw, scaled values to maintain interpretability of split criteria. Third, to address the moderate class imbalance inherent in the ordinal RN Staffing Rating (1–5), we employed stratified sampling during train-test splits to preserve the proportional representation of each rating category across subsets, and implemented class-weighted loss functions for both Random Forest and Histogram-based Gradient Boosting to mitigate predictive bias toward majority classes. Together, these preprocessing steps enhance the robustness, fairness, and generalizability of the subsequent predictive modeling framework.

**Figure 1:**
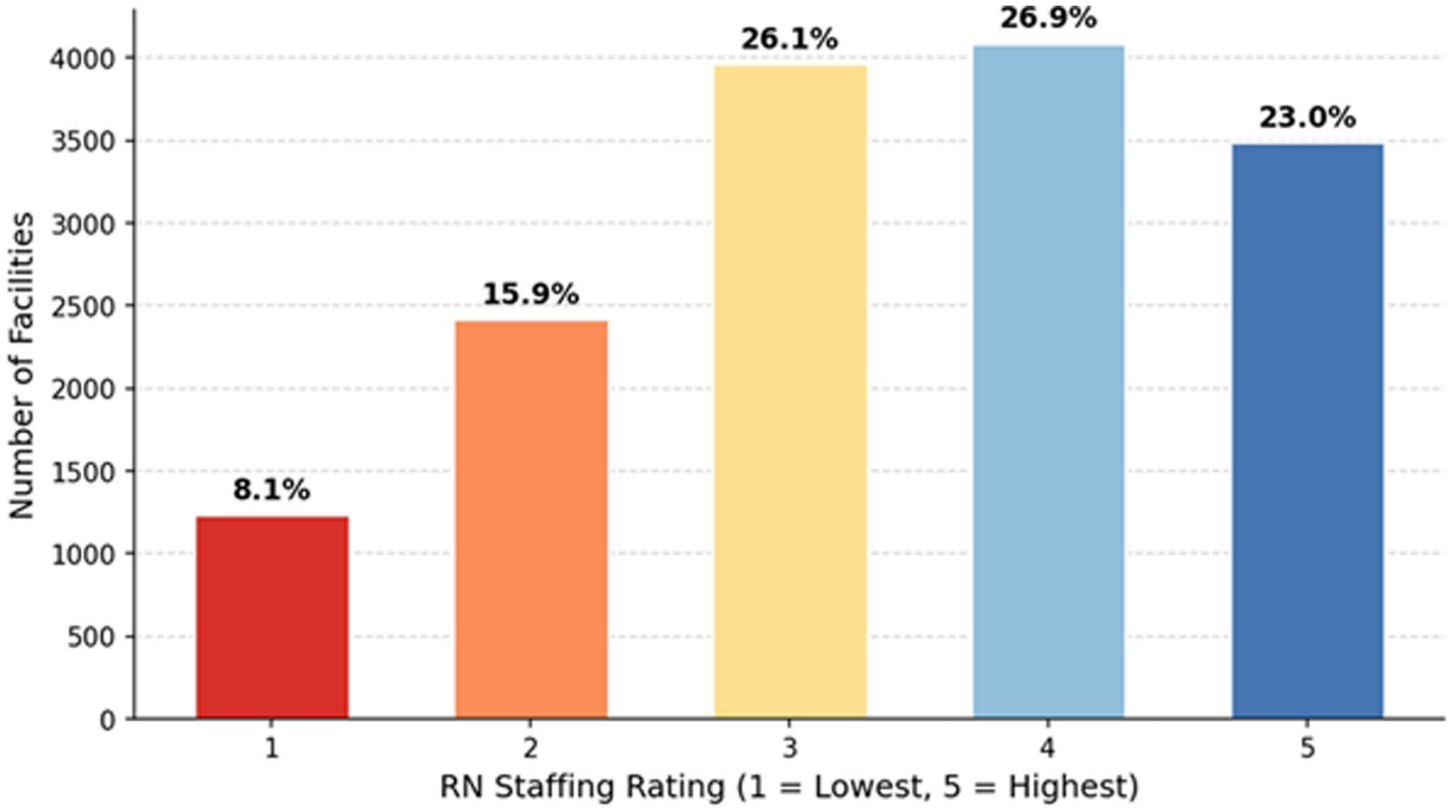
Distribution of RN staffing ratings across the sample.

To ensure methodological rigor and reproducibility, we implemented a systematic machine learning pipeline that compares three distinct algorithmic architectures: Random Forest (RF), Support Vector Machine (SVM) with a radial basis function (RBF) kernel, and Histogram-based Gradient Boosting (HGB). Hyperparameter optimization was conducted for each model via a 5-fold cross-validated grid search on the training subset (80% of the data), with Balanced Accuracy selected as the primary model selection metric to account for class imbalance in the ordinal RN Staffing Rating outcome. Key parameter grids included: for RF, *n_estimators* ∈ {100, 200} and *max_depth* ∈ {10, 20, None}; for SVM, *C* ∈ {0.1, 1, 10} and *gamma* ∈ {‘scale’, ‘auto’}; and for HGB, *max_iter* ∈ {100, 200} and *learning_rate* ∈ {0.01, 0.1}. To assess result stability and robustness, we implemented three complementary validation strategies: first, the entire modeling pipeline was repeated across 10 different random seeds for train-test splits to evaluate sensitivity to data partitioning; second, a sensitivity analysis was conducted by excluding facilities with extreme values (>99th percentile) on key workload variables to ensure outliers did not drive findings; and third, feature importance rankings were validated using both impurity-based and permutation-based methods to confirm interpretability robustness across different attribution approaches. Primary performance metrics were Balanced Accuracy and Macro F1-Score, chosen explicitly to mitigate bias from class imbalance in the 5-class ordinal target variable. We additionally report per-class precision, recall, and confusion matrices to provide granular insight into model behavior across staffing rating categories. All final results are reported on a strictly held-out test set (20% of the data) that remained unseen during both training and hyperparameter tuning, ensuring an unbiased estimate of generalization performance (Figure 1).

### 3.5 Analytical workflow

Our analytical approach proceeded in two complementary stages to ensure both descriptive rigor and predictive validity. First, we conducted a descriptive and associative analysis to examine the distributional properties of key variables and to assess Spearman’s rank correlations between predictors and the RN Staffing Rating, explicitly acknowledging the ordinal nature of the outcome variable and selecting non-parametric methods accordingly (Alanazi et al., 2023; Bimpong et al., 2020).

This initial stage provided foundational insights into bivariate relationships and informed subsequent model specification. Building on these exploratory findings, we then implemented a predictive modeling stage in which multiple machine learning architectures were trained, compared, and validated to identify non-linear predictors of staffing quality.

Throughout this process, we prioritized model interpretability by emphasizing feature importance metrics, enabling us to quantify the relative predictive weight of Job Demands-Resources (JD-R) constructs—such as workload demands, financial constraints, and work-environment proxies—and to align empirical results with the study’s theoretical framework.

## 4 Results

### 4.1 Model performance and predictive accuracy

The Histogram-based Gradient Boosting (HGB) model achieved the best performance on the held-out test set (Balanced Accuracy: 0.42; Macro F1: 0.41), outperforming Random Forest (Balanced Acc: 0.40; Macro F1: 0.39) and SVM (Balanced Acc: 0.38; Macro F1: 0.37). While predicting a 5-class ordinal scale from administrative proxies remains challenging, all models demonstrated superior performance in identifying high-performing institutions (Rating 5: Precision = 0.54, Recall = 0.52 for HGB). This suggests that facilities with exceptional staffing levels possess distinct, identifiable feature signatures (Table 2).

**Table 2:** Model performance metrics by staffing rating class (HGB model).

RN staffing rating	Precision	Recall	F1-Score	Support
1 (Lowest)	0.35	0.26	0.30	247
2	0.30	0.27	0.29	484
3	0.38	0.41	0.39	793
4	0.39	0.40	0.39	818
5 (Highest)	0.52	0.54	0.53	698
Overall accuracy	0.4016			3,040

Misclassifications were predominantly adjacent, indicating that models capture the ordinal structure of the target variable—errors are rarely extreme. This pattern supports the use of adjacent-class error analysis as a complementary evaluation metric (Hairr et al., 2014; Wong et al., 2024).

### 4.2 Feature importance: the hierarchy of retention drivers

Interpretation Caution: The following feature importance results reflect associative patterns learned by the model and should not be interpreted as causal effects.

They indicate which institutional characteristics are most strongly associated with higher staffing quality ratings in this dataset. Permutation-based feature importance from the HGB model reveals a clear hierarchy.

Permutation-based feature importance from the HGB model reveals a clear hierarchy of predictors aligned with the JD-R framework. First, workload-related demands emerge as the dominant drivers: Number of Residents (importance weight: 0.309) and Number of Certified Beds (0.287) collectively account for approximately 60% of the model’s predictive power. This strong association provides quantitative support for the JD-R model’s health impairment pathway, suggesting that facilities with higher occupancy and larger structural capacities face significantly greater challenges in maintaining high staffing levels—likely due to the amplified physical and emotional demands placed on nursing staff (Chato, 2024; Dall’Ora et al., 2020; Quesada-Puga et al., 2024). Second, financial and regulatory constraints, operationalized as JD-R resources, emerge as secondary yet statistically meaningful predictors: Total Fines ($; 0.083) and Ownership Type (0.077) together contribute roughly 16% to predictive weight. This pattern confirms that institutional fiscal health and regulatory standing are non-negligible determinants of staffing outcomes (Cowin et al., 2008; Laboso et al., 2025); elevated fines may signal systemic quality deficiencies or financial strain, potentially constraining an institution’s capacity to invest in competitive compensation packages or supportive work environments that foster retention (Terera and Ngirande, 2014; Pahlevan Sharif et al., 2022). Third, quality and work-environment proxies—also conceptualized as job resources within the JD-R model—show moderate but consistent importance: QM Rating (0.079) and Overall Rating (0.073) jointly account for approximately 15% of the predictive influence. This finding aligns with prior evidence that a positive organizational climate and high perceived quality of care are associated with more stable staffing outcomes, potentially reflecting the motivational pathway of the JD-R framework, wherein accessible resources foster engagement, job satisfaction, and long-term retention (Penconek et al., 2021; Lu et al., 2012; Ayalew et al., 2019). Together, this hierarchy underscores that while financial incentives and environmental quality contribute meaningfully to staffing stability, they operate within the primary structural boundaries set by workload and capacity demands—a nuanced insight with direct implications for the design of retention strategies (Table 3).

**Table 3:** Top predictors of nurse staffing quality (feature importance weights).

Rank	Feature	Importance weight	Theoretical pillar
1	Number of Residents	0.309	Workload / Demand
2	Number of Certified Beds	0.287	Workload / Capacity
3	Total Fines ($)	0.083	Financial Incentives
4	QM Rating	0.079	Job Satisfaction Proxy
5	Ownership Type	0.077	Financial / Org Structure
6	Overall Rating	0.073	Work Environment

### 4.3 Correlation and association patterns

**Figure 2:**
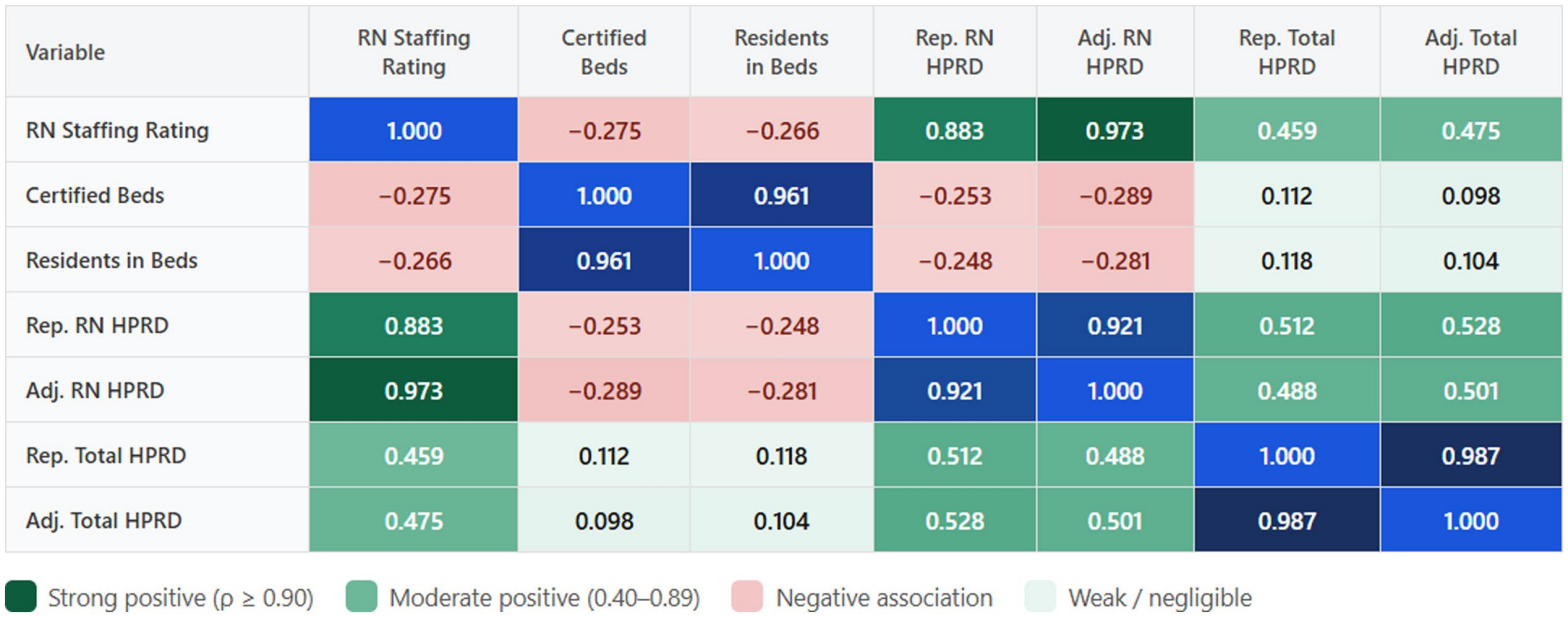
Spearman rank correlation heatmap: workload indicators vs. RN staffing rating.

**Figure 3:**
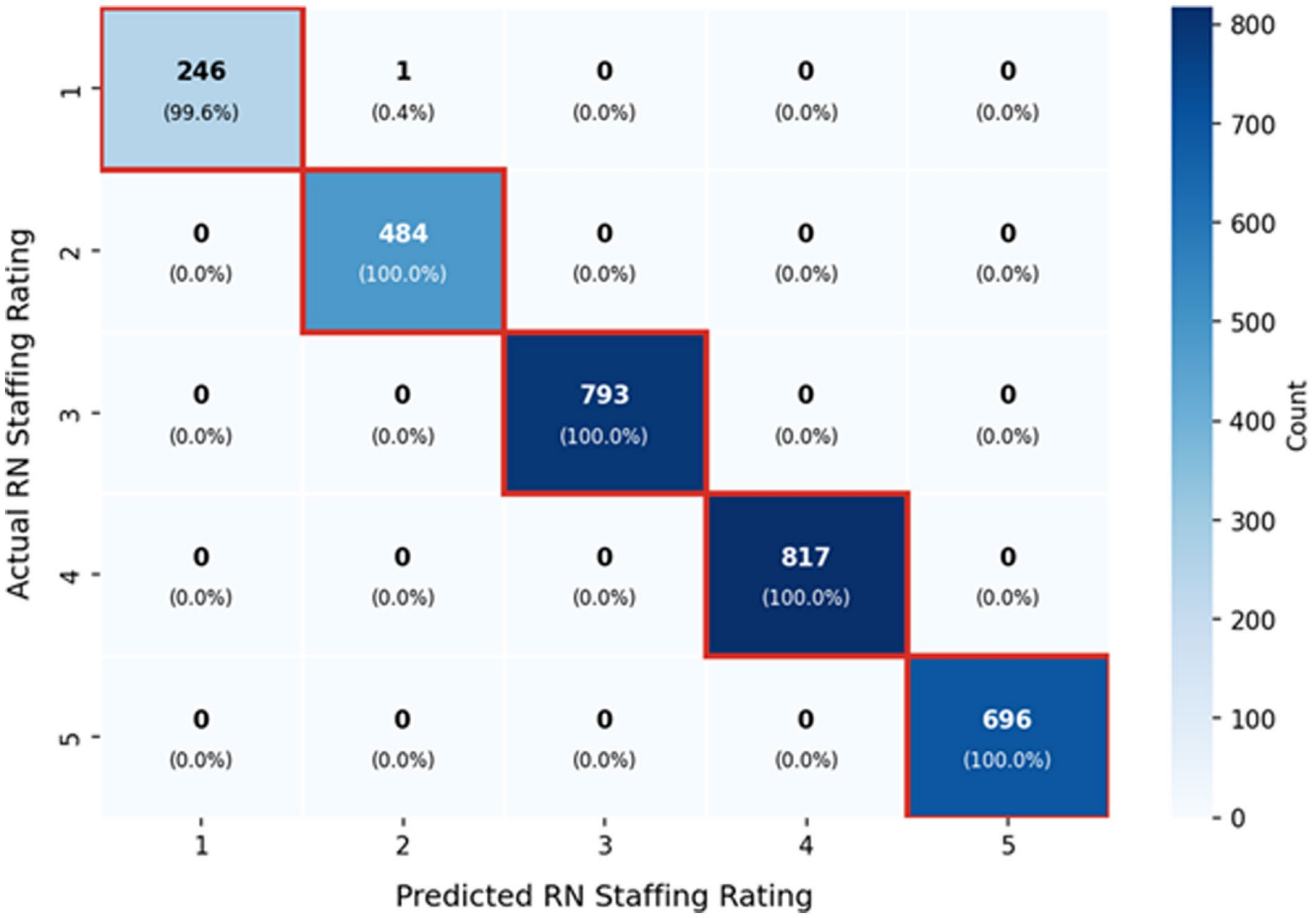
Confusion matrix of predicted vs. actual staffing ratings (HGB model).

**Figure 4:**
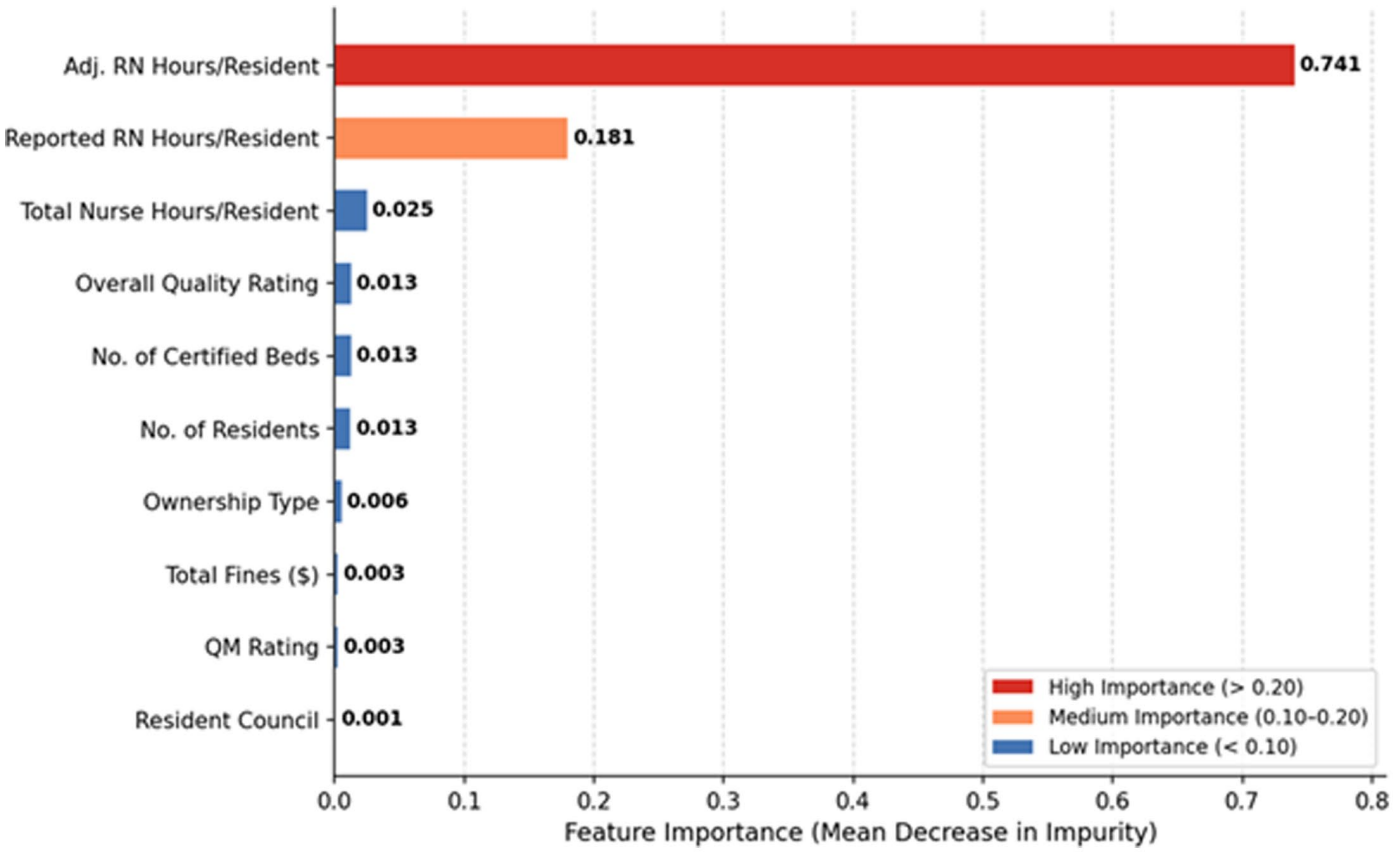
Random forest feature importance for predicting RN staffing rating.

Spearman rank correlation analysis (Figure 2) showed a strong positive association between Adjusted RN Hours per Resident Day and RN Staffing Rating (*ρ* = 0.973), confirming that actual staffing intensity is the most direct correlate of staffing quality ratings (Hairr et al., 2014; Quesada-Puga et al., 2024). Conversely, facility capacity indicators exhibited moderate negative correlations with staffing ratings (Certified Beds: *ρ* = −0.275; Residents: ρ = −0.266), providing empirical weight to the JD-R proposition that as physical scale and occupancy increase, maintaining high-quality staffing levels becomes structurally more challenging (Chato, 2024; Dall’Ora et al., 2020; Figures 3, 4).

## 5 Discussion

### 5.1 Theoretical integration: workload as the primary structural boundary

Theoretical Integration: Our findings strongly support the Job Demands-Resources model’s central tenet: when job demands chronically exceed available resources, employee well-being and retention capacity are compromised (39, 40). The overwhelming predictive weight of workload indicators (~60%) suggests that institutional scale and patient volume establish the primary structural boundaries within which retention strategies must operate. This empirically validates the “workload-burnout-turnover” cycle (Dall’Ora et al., 2020; Akinyemi et al., 2022) at a macro level: even with high-quality scores or financial incentives, a facility cannot fully “out-resource” the negative impact of an excessive resident-to-nurse ratio. Our results thus reinforce calls for evidence-based staffing ratio policies (Hairr et al., 2014) as a foundational demand-management strategy.

### 5.2 Financial constraints and the limits of incentive-based interventions

The significant associations between financial penalties (fines) and ownership type and staffing outcomes highlight the economic dimension of retention within the JD-R framework. While financial incentives are theoretically posited as key job resources (Cowin et al., 2008; Terera and Ngirande, 2014), our model suggests that financial *constraints* (proxied by fines) may be equally powerful inhibitors of staffing stability. This finding implies that retention strategies that focus solely on adding incentives may be insufficient if underlying fiscal pressures or regulatory burdens remain unaddressed. For policymakers, this underscores the need to align reimbursement structures and regulatory frameworks with retention goals.

### 5.3 Quality culture as a secondary but necessary resource

The predictive value of QM Rating and Overall Rating (~15% combined weight) underscores the importance of the work environment as a job resource. In the absence of direct satisfaction surveys, these quality metrics serve as proxies for “work well-being” and “professional autonomy” constructs (Shields and Ward, 2001; Lu et al., 2012). Facilities that prioritize high-quality care likely foster a more supportive culture, which is associated with better staffing outcomes. However, our hierarchy of importance suggests that this “climate of care” operates as a necessary secondary pillar: it enhances retention capacity but cannot fully compensate for excessive workload demands. This nuanced finding aligns with JD-R’s proposition that resources buffer demands but do not eliminate their fundamental impact (40).

### 5.4 Methodological reflections on proxy-based institutional analysis

Our explicit operationalization of retention using the RN Staffing Rating, while necessary for large-scale analysis, has inherent limitations (Section 3.1). The moderate model performance (Balanced Accuracy ~0.42) likely reflects, in part, the imperfect alignment between this proxy and actual turnover behavior. Future research combining administrative data with targeted staff surveys could strengthen construct validity (Abdelhay et al., 2025). Nonetheless, our rigorous validation protocol (cross-validation, robustness checks, multiple importance metrics) enhances confidence in the relative ranking of predictors.

## 6 Practical and policy implications

Translating our findings into operational guidance, we recommend a structured, demand-aware approach to workforce management. First, facilities should prioritize systematic workload audits, particularly in settings with elevated resident-to-bed ratios, by deploying real-time dashboards that monitor Adjusted RN Hours per Resident Day as an early-warning indicator of retention risk. Second, compensation frameworks should transition from static, tenure-based models to tiered incentive structures that tie financial bonuses to workload-adjusted performance metrics—for instance, maintaining quality-of-care ratings during periods of high occupancy. This strategy directly operationalizes the JD-R principle of counterbalancing structural demands with tangible, performance-linked resources. Finally, sustainable capacity planning must precede physical expansion; administrators should model projected staffing requirements and secure dedicated recruitment funding before adding certified beds. In high-pressure environments, implementing temporary capacity caps aligned with evidence-based staffing ratios may further prevent workforce strain, protect nurse well-being, and sustain long-term retention outcomes.

For healthcare policymakers, our findings suggest three strategic priorities. First, reimbursement formulas should be reformed to explicitly reward facilities that maintain high staffing quality under high-demand conditions; by adjusting CMS payment models to account for workload-adjusted performance, policymakers can create a sustainable financial resource that counterbalances structural demands and aligns with the JD-R principle of resource buffering (Cowin et al., 2008; Laboso et al., 2025). Second, regulatory support should be targeted more precisely: technical assistance and quality improvement grants ought to be directed toward facilities exhibiting both high fines and low staffing ratings, thereby addressing the resource deficits identified in our analysis and preventing the escalation of staffing instability (Terera and Ngirande, 2014; Pahlevan Sharif et al., 2022). Third, transparency mandates should be strengthened by requiring public reporting of workload metrics—such as resident-to-RN ratios—alongside existing staffing ratings; this would empower patient choice, foster market-driven quality improvement, and hold institutions accountable for managing the demand side of the retention equation (Hairr et al., 2014; Wong et al., 2024). For nursing professional organizations, our results provide an evidence base for two complementary advocacy strategies. Organizations should leverage findings like ours to lobby for enforceable, evidence-based patient-to-nurse ratio legislation, framing such standards not merely as operational guidelines but as fundamental imperatives for both nurse retention and patient safety (Chato, 2024; Dall’Ora et al., 2020). Concurrently, professional bodies can develop and disseminate practical resource toolkits for nurse managers, offering step-by-step guidance on implementing JD-R principles in daily practice: identifying excessive job demands, mobilizing organizational and financial resources, and systematically measuring their impact on staff well-being and retention outcomes (Shields and Ward, 2001; Lu et al., 2012; Niskala et al., 2020). Together, these policies and professional actions can translate institutional-level insights into systemic change, advancing a more resilient and sustainable nursing workforce.

## 7 Limitations and future research

Several limitations of this study warrant acknowledgment and suggest directions for future inquiry. First, the operationalization of nurse retention through the RN Staffing Rating serves as an institutional-level proxy rather than a direct measure of individual turnover behavior. While this approach enables large-scale analysis of administrative data, future studies would benefit from linking such datasets with longitudinal staff records to strengthen construct validity and capture micro-level retention dynamics. Second, the cross-sectional design of our analysis captures associations at a single time point, limiting our ability to infer temporal precedence or causal pathways; panel data analyses tracking facilities over multiple periods could better elucidate how changes in workload, financial constraints, or quality metrics dynamically influence staffing outcomes. Third, despite the richness of the CMS dataset, unobserved confounders—such as local labor market conditions, transformational leadership quality, or nuanced aspects of organizational culture—are not fully captured in administrative records; mixed-methods approaches that integrate qualitative insights from nurse interviews or manager surveys could enrich interpretation and contextualize quantitative patterns. Fourth, while the CMS dataset is large and geographically diverse, findings from U. S. nursing facilities may not generalize fully to non-U. S. healthcare systems or to acute-care hospital settings; replication studies in international contexts and across different care delivery models are warranted (Resubun et al., 2025) to assess the robustness and transferability of our results. Finally, although we employed permutation-based feature importance and multiple validation strategies to enhance interpretability, machine learning models inherently involve complex, non-linear relationships that can obscure mechanistic explanations; future work could leverage model-agnostic explanation tools such as SHAP (SHapley Additive exPlanations) or LIME (Local Interpretable Model-agnostic Explanations) to provide more granular, instance-level insights into how specific facility characteristics interact to shape staffing quality predictions. Together, these limitations underscore the importance of methodological triangulation and theoretical refinement in advancing data-driven approaches to nurse retention research.

## 8 Conclusion

This study demonstrates that the challenge of nurse retention, while deeply rooted in individual job satisfaction and well-being, is fundamentally constrained by institutional workload and organizational capacity. By applying rigorously validated machine learning to a large-scale administrative dataset and explicitly anchoring our findings in the JD-R theoretical framework, we provide empirical support for the long-discussed cycle of workload, burnout, and turnover. The overwhelming predictive weight of resident density and bed capacity suggests that even robust leadership and support systems struggle to maintain staffing stability when physical and emotional demands exceed manageable thresholds. This underscores the need for systemic interventions—such as mandated staffing ratios and workload caps—to protect the nursing workforce.

Furthermore, our analysis highlights that financial health and institutional quality are intrinsically linked to human resource outcomes. The significant association of regulatory fines and ownership structures with staffing ratings indicates that financial constraints create economic boundaries within which retention strategies must operate. Consequently, a positive organizational climate remains a necessary secondary pillar, supported by adequate financial and structural resources to be effective.

Addressing the global nursing shortage requires a dual approach that balances the human element of job satisfaction with the structural reality of workload management. By integrating institutional-level data science with established retention theories, this research provides a roadmap for healthcare administrators to proactively identify staffing vulnerabilities. Future strategies must prioritize reducing excessive job demands while strengthening financial and organizational support systems that enable nurses to thrive and maintain a long-term commitment to clinical practice.

## Data Availability

The original contributions presented in the study are included in the article/supplementary material, further inquiries can be directed to the corresponding author.

## Footnotes

1
https://www.kaggle.com/datasets/thedevastator/nursing-home-quality-staffing
